# Editorial: Breastfeeding: a sociological reflection on new mothers' wellbeing

**DOI:** 10.3389/fsoc.2023.1291140

**Published:** 2023-09-26

**Authors:** Sarah Earle, Alison Spiro

**Affiliations:** ^1^Faculty of Wellbeing, Education & Language Studies, The Open University, Milton Keynes, United Kingdom; ^2^Department of Life Sciences, Brunel University London, London, United Kingdom

**Keywords:** breastfeeding, lactation, infant feeding, woman centered care, sociology of health

In this editorial, we consider a sociological reflection on breastfeeding and new mother's wellbeing, a Research Topic that was launched to celebrate World Breastfeeding Week. This is a global campaign, which began in 1992, designed to raise awareness and action on breastfeeding.

There are six original research articles and one case report within this Research Topic. We briefly outline each of these contributions below.

In the first article in this Research Topic, Thomson et al., explore why many women discontinue breastfeeding in the early post-natal period, even when they start out with strong intentions to breastfeed. They draw on a secondary analysis of 24 interviews with women; a sub-set of data collected from the Assets-based feeding help Before and After birth (ABA) feasibility trial (see Clarke et al., 2020) in the UK. Using a complex-systems lens approach, the authors explore how a range of factors including the individual, mother-infant dyad, health services, family and social networks, as well as wider community infrastructure, interact with women's motivations and experiences of breastfeeding. Two key infant feeding typologies are presented: “disappointed” women stopped breastfeeding early, and “by hook or by crook” women continued breastfeeding despite facing challenges.

In the second article, Zavala-Soto et al., explore how cesarean section surgery can be modified—without requiring structural changes in health facilities or additional staff resource—to encourage skin-to-skin contact, to promote maternal satisfaction and breastfeeding. The authors draw on a qualitative research study in Mexico, which has a high rate of cesarean sections and one of the lowest prevalence of exclusive breastfeeding in Latin America. Using a grounded theory approach, the authors deployed a qualitative survey, recruiting 150 women, followed by 36 face-to-face intensive interviews. The research study found that several factors influenced breastfeeding success including: intention to breastfeed, maternal care during birth, the encouragement of skin-to-skin contact and co-housing, and professional support postnatally.

The article by Palmquist et al., reports on the outcomes of an online survey on human milk feeding practices carried out during the first wave of the COVID-19 pandemic in the United States. The U.S. is the only high-income country that does not federally mandate protection of postpartum employment through paid postpartum maternity and family leave policies. The authors describe their research study—based on 1,437 eligible survey responses—as a “natural experiment” investigating the impact of *de facto* paid leave on infant feeding practices after the enactment of stay-at-home orders. Although the COVID-19 pandemic created unprecedented challenges for women and families with infants, the research found immediate positive effects of stay-at-home policies on human milk feeding practices. The authors conclude that federally mandated paid postpartum and family leave are essential to achieving more equitable lactation outcomes.

The next article, written by Quinones, presents an autoethnographic account of breastfeeding in northern Spain. In this—using personal vignettes—the author reports on her own experience of first-time motherhood and infant feeding within the context of the feminist and other literature. The article exposes the consequences of competing breastfeeding discourses which—on the one hand—position breastfeeding as natural and easy—while on the other—relying on medical practices and interventions when difficulties arise. The author concludes that the representation of breastfeeding as unproblematic within breastfeeding campaigns is harmful to women and babies and should better reflect the diversity and realities of experience.

In the fifth article Augusto et al. also examine the way in which breastfeeding is both a natural, physiological process as well as a social practice subject to strong social expectations and moral judgements. Drawing on an exploratory qualitative study carried out in Portugal using focus group methods, the article explores five dimensions of breastfeeding experience (1) the decision to breastfeed (2) expertise and information (3) conciliation (4) the role of the father, and (5) self and identity. The article highlights how experiences of breastfeeding can only be understood when situated within the context of women's lives. It also shows that breastfeeding can be viewed by women as an empowering experience.

In the next article, Roberts et al., shift their attention to the experiences of women that formula feed their babies in the UK, a country which has very low rates of exclusive breastfeeding at six-months following birth. The article draws on an online study of 624 mothers which sought to investigate infant feeding experiences through open text survey responses. Content analysis of the survey data identified four clusters of reasons for formula feedgin: (1) feeding attitudes (2) feeding problems (3) mental health, and (4) sharing the load. On the basis of their findings the authors make several recommendations to improve breastfeeding education, including the refinement of practical breastfeeding skills, the training of mental health practitioners on infant feeding, and the tightening of workplace legislation to protect breastfeeding women.

The final contribution to this Topic is a case report on breastfeeding grief following masculinization mastectomy and (de)transition written by Gribble et al.. This deals sensitively with an important and complex subject highlighting the lack of attention paid to breast function within transgender surgical literature and the subsequent lack of understanding within maternity services of the significance of human milk feeding for women with mastectomies. The authors call for honest, knowledgeable and compassionate individualized support for new mothers without functioning breasts. They recommend that breastfeeding organizations, together with surgical associations, gender identity services, and transgender guideline producers work collaboratively to recognize the importance of breast function and breastfeeding when addressing the needs of those who seek, or have had, masculinization breast/chest surgery.

In this Research Topic we have explored a number of long-standing, as well as more contemporary concerns on breast-feeding and new mothers' wellbeing. The articles have used a wide range of methodological approaches and theoretical frameworks, while focusing on the importance of placing women's experience at the center of analysis. Most of the articles highlight the complex nature of breastfeeding, as well as the multiple and competing discourses within which breastfeeding is enacted and practiced around the world.

## Author contributions

SE: Writing—original draft. AS: Writing—review and editing.
